# 532. Nirmatrelvir/Ritonavir Versus Placebo in Unvaccinated and Vaccinated High Risk Patients

**DOI:** 10.1093/ofid/ofad500.601

**Published:** 2023-11-27

**Authors:** Heidi Leister-Tebbe, Weihang Bao, Robert Fountaine, Mary Lynn Baniecki, Victoria Hendrick, Wayne Wisemandle, Jennifer Hammond, James Rusnak

**Affiliations:** Pfizer Inc, Collegeville, Pennsylvania; Pfizer Inc, Collegeville, Pennsylvania; Pfizer Inc, Collegeville, Pennsylvania; Pfizer Inc, Collegeville, Pennsylvania; Pfizer, Sandwich, England, United Kingdom; Pfizer Inc, Collegeville, Pennsylvania; Pfizer Inc, Collegeville, Pennsylvania; Pfizer Inc, Collegeville, Pennsylvania

## Abstract

**Background:**

Nirmatrelvir with ritonavir (nirmatrelvir/r) is an oral antiviral treatment for COVID-19. EPIC-high risk (HR) was a Ph 2/3 double-blind, randomized, placebo (PBO)-controlled trial to evaluate nirmatrelvir/r in symptomatic, unvaccinated, nonhospitalized patients (pts) with ≥ 1 risk factor for progression to severe COVID-19. EPIC-standard risk (SR), a similarly designed trial enrolled pts with no risk factors, and fully vaccinated pts with ≥ 1 risk factor. An integrated analysis of treated pts with risk factors, including unvaccinated pts from EPIC-HR and vaccinated pts from EPIC-SR has been conducted.

**Methods:**

Eligible adults from EPIC-HR and EPIC-SR with confirmed SARS-CoV-2 were randomized 1:1 within 5 days (d) of symptom onset to receive nirmatrelvir/r 300 mg/100 mg or PBO every 12 hrs for 5 d. Efficacy of nirmatrelvir/r was compared with PBO for COVID-19-related hospitalization or death from any cause through day 28. Other endpoints, including number of medical visits/hospital days and quantification of SARS-CoV-2 viral RNA load in nasopharyngeal swabs, and TEAEs through day 35 were evaluated.

**Results:**

Nirmatrelvir/r reduced COVID-19-related hospitalization or death due to any cause by 83% (Relative Risk Reduction, RRR) compared to the PBO (0.94% vs 5.52%, p< 0.0001). The rate of hospitalization or death was lower among sero+ or vaccinated pts, however, nirmatrelvir/r treatment was associated with a substantial risk reduction among sero+ EPIC-HR pts (87.9% RRR), vaccinated EPIC-SR pts (57.6% RRR), or pts who were either vaccinated or sero+ (73.8% RRR) (Figure 1). These observations were regardless of age, gender, BMI, coexisting conditions, baseline serology status, viral RNA load and vaccination status (Figure 2). On average, pts treated with nirmatrelvir/r spent significantly fewer days in hospital and had significantly fewer medical visits compared with PBO. Nirmatrelvir/r treated pts had significantly greater reductions in viral RNA load from baseline to day 5 than PBO. Rates of all-causality TEAEs were similar between treatment groups and most were mild to moderate in severity.

**Figure:**
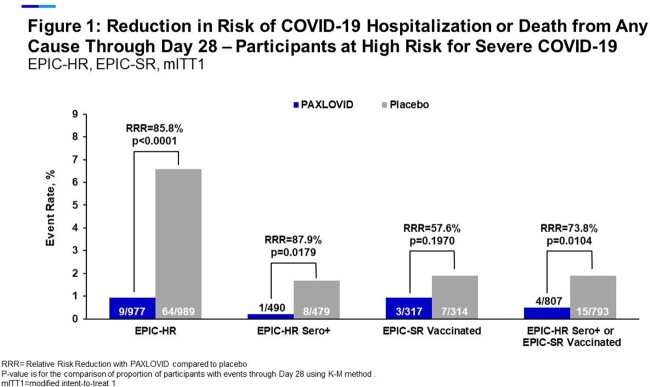

**Figure:**
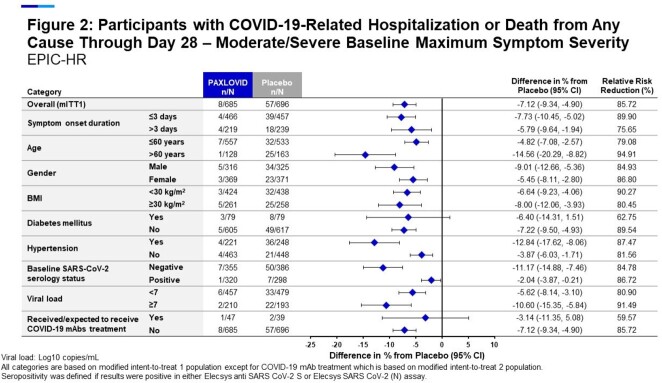

**Conclusion:**

The efficacy and safety of nirmatrelvir/r was demonstrated in vaccinated and unvaccinated pts at high risk as well as in pts with serologic evidence of prior exposure to COVID-19.

**Disclosures:**

**Heidi Leister-Tebbe, BSN**, Pfizer Inc: Employee|Pfizer Inc: Stocks/Bonds **Weihang Bao, PhD**, Pfizer, Inc.: Employee of Pfizer|Pfizer, Inc.: Stocks/Bonds **Robert Fountaine, PharmD**, Pfizer Inc: Employee|Pfizer Inc: Stocks/Bonds **Mary Lynn Baniecki, PhD**, Pfizer Inc: Employee|Pfizer Inc: Stocks/Bonds **Victoria Hendrick, BSc**, Pfizer: Employee|Pfizer: Stocks/Bonds **Wayne Wisemandle, MA**, Pfizer Inc: Employee|Pfizer Inc: Stocks/Bonds **Jennifer Hammond, PhD**, Pfizer: Employee|Pfizer: Stocks/Bonds **James Rusnak, MD, PhD**, Pfizer Inc: Employee|Pfizer Inc: Stocks/Bonds

